# Relations between personal exposure to elevated concentrations of arsenic in water and soil and blood arsenic levels amongst people living in rural areas in Limpopo, South Africa

**DOI:** 10.1007/s11356-023-26813-9

**Published:** 2023-04-20

**Authors:** Thandi Kapwata, Caradee Y. Wright, Tarylee Reddy, Renee Street, Zamantimande Kunene, Angela Mathee

**Affiliations:** 1 Environment and Health Research Unit, South African Medical Research Council, Johannesburg, 2028 South Africa; 2 Environmental Health Department, Faculty of Health Sciences, University of Johannesburg, Johannesburg, 2028 South Africa; 3 Environment and Health Research Unit, South African Medical Research Council, Pretoria, 0084 South Africa; 4 Department of Geography, Geoinformatics and Meteorology, University of Pretoria, Pretoria, 0001 South Africa; 5 Biostatistics Research Unit, South African Medical Research Council, Durban, 4001 South Africa; 6 School of Mathematics, Statistics and Computer Science, University of KwaZulu Natal, Pietermaritzburg, 3201 South Africa; 7 Environment and Health Research Unit, South African Medical Research Council, Durban, 4001 South Africa; 8 School of Public Health, University of the Witwatersrand, Johannesburg, 2028 South Africa

**Keywords:** Biomarker, Environmental health, Environmental exposure, Metals, Risk, Vulnerability

## Abstract

Exposure to arsenic even at low levels can lead to adverse health outcomes, however, there is a paucity of research from South Africa in relation to human exposure to arsenic. We investigated long-term exposure of residents in Limpopo province, South Africa, in a cross-sectional study by analysing water, soil and blood arsenic concentrations from two arsenic-exposed (high and medium–low exposure) villages and one non-exposed (control) village. There were statistically significant differences in the distribution of arsenic in water, soil and blood amongst the three sites. The median drinking water arsenic concentration in the high-exposure village was 1.75 µg/L (range = 0.02 to 81.30 µg/L), 0.45 µg/L (range = 0.100 to 6.00 µg/L) in the medium- / low-exposure village and 0.15 µg/L (range =  < limit of detection (LOD) to 29.30 µg/L) in the control site. The median soil arsenic concentration in the high-exposure village was 23.91 mg/kg (range =  < LOD to 92.10 mg/kg) whilst arsenic concentrations were below the limit of detection in all soil samples collected from the medium-/low-exposure and control villages. In the high-exposure village, the median blood arsenic concentration was 1.6 µg/L (range = 0.7 to 4.2 µg/L); 0.90 µg/L (range =  < LOD to 2.5 µg/L) in the medium-/low-exposure village and 0.6 µg/L (range =  < LOD to 3.3 µg/L) in the control village. Significant percentages of drinking water, soil and blood samples from the exposed sites were above the internationally recommended guidelines (namely, 10 µg/L, 20 mg/kg and 1 µg/L, respectively). Majority of participants (86%) relied on borehole water for drinking and there was a significant positive correlation between arsenic in blood and borehole water (*p*-value = 0.031). There was also a statistically significant correlation between arsenic concentrations in participants’ blood and soil samples collected from gardens (*p*-value = 0.051). Univariate quantile regression found that blood arsenic concentrations increased by 0.034 µg/L (95% CI = 0.02–0.05) for each one unit increase in water arsenic concentrations (*p* < 0.001). After adjusting for age, water source and homegrown vegetable consumption in multivariate quantile regression, participants from the high-exposure site had significantly higher blood concentrations than those in the control site (coefficient: 1.00; 95% CI = 0.25–1.74; *p*-value = 0.009) demonstrating that blood arsenic is a good biomarker of arsenic exposure. Our findings also provide new evidence for South Africa on the association between drinking water and arsenic exposure, emphasising the need for the provision of potable water for human consumption in areas with high environmental arsenic concentrations.

**Supplementary Information:**

The online version contains supplementary material available at 10.1007/s11356-023-26813-9.

## Introduction

Chronic exposure to arsenic is a significant environmental public health concern. The growing body of evidence suggests adverse health outcomes occur even at low levels of exposure (NRC 2014; Yang et al. 2021; Moon et al. 2017). Arsenic exposure affects several body organs leading to ill-health outcomes including skin lesions, neurological impairments, respiratory and cardiovascular disorders, and disruptions to immune and endocrine systems (Naujokas et al. 2013). Arsenic is classified as a Group 1 human carcinogen by the International Agency for Research on Cancer (IARC 2004) with exposure to elevated arsenic concentrations associated with increased risk of several human cancers (IARC 2012). Epidemiological studies suggest that major health risks from arsenic are due to chronic exposure to contaminated drinking water and soil. However, water is deemed the predominant source of arsenic. Several clinical and epidemiological studies conducted globally have assessed the health impacts of exposure to arsenic-contaminated drinking water (Chen et al. 2011; Ferreccio et al. 2013; Lamm et al. 2013; Liaw et al. 2008; Monrad et al. 2017). Arsenic-endemic areas, such as Bangladesh in India, the southwest coast of Taiwan, and parts of Chile, have reported elevated risks and prevalence of skin, bladder, liver, kidney and prostate cancer due to exposure to arsenic-contaminated drinking water (Steinmaus et al. 2013; Smith et al. 2000; Chen and Ahsan 2004; Chen 2014; Cheng et al. 2016; Kumar et al. 2021).

A predominant clinical manifestation of arsenic toxicity is black-foot disease. This is a peripheral vascular disease associated with long-term exposure to inorganic arsenic that is common in areas with elevated arsenic concentrations in groundwater used for human consumption (Nordberg et al. 2019). Increased risk and prevalence of cardiovascular diseases, such as myocardial infarction and diabetes, have also been associated with human consumption of arsenic-contaminated drinking water (D’Ippoliti et al. 2015; Chen et al. 2011; Tseng 2008; Del Razo et al. 2011). Neurologic examinations and assessments conducted on study participants exposed to high and even low concentrations of arsenic in drinking water in India (Mukherjee et al. 2003; Chakraborti et al. 2003), Taiwan (Tsai et al. 2003; Tseng et al. 2006), the United States of America (Wasserman et al. 2014) and Myanmar (Mochizuki et al. 2019) demonstrate varying types and severity of arsenic-typical neuropathy. Prolonged exposure to arsenic has also been found to reduce cognitive ability, especially in children (Nahar et al. 2014; Dong and Su 2009; Tolins et al. 2014). These studies provide evidence of the health risks of arsenic exposure from consuming ground water in several parts of the world. However, there is a scarcity of similar research in South Africa, where the majority of research is centred around quantifying environmental arsenic concentrations, with little focus on health effects. These include studies that have assessed arsenic contamination of soil (Kapwata et al. 2020; Kootbodien et al. 2012; Mathee et al. 2018) and water sources (Mudzielwana et al. 2020; Genthe et al. 2018; Munyangane et al. 2017) where results found that concentrations of arsenic were above international guidelines. An environmental study conducted in villages in Giyani, Limpopo province, found that 54% out of a total of 95 soil samples collected in one village had arsenic concentrations exceeded the Canadian Soil Quality Guidelines for the Protection of Environmental and Human Health of 20 mg/kg (CEPA 2007) which considers lifetime cancer risk due to exposure. Unfortunately, there is no similar South African guideline to apply. The associated pollution index, which was calculated to categorise the level of arsenic contamination, determined that 57% of those samples were classified as ‘moderately to heavily’ and ‘extremely’ contaminated (Kapwata et al. 2020). Whilst a South African guideline limit for arsenic in residential soil of 47 mg/kg exists, it is based on soil contamination, not on evidence of adverse health outcomes. Therefore, Kapwata et al. (2020) applied the Canadian guideline, because it is based on adverse effects on humans and the environment. Other studies (Mudzielwana et al. 2020; Munyangane et al. 2017), have analysed arsenic concentrations in drinking water samples from boreholes in Giyani (including water samples from schools) and found concentrations that surpassed the limit of 10 µg/L recommended by both the South African and the World Health Organization (WHO) guidelines for drinking water quality (SANS 2015; WHO 2017).

Several South African studies have conducted arsenic health risk assessments and characterised the risks of personal or community exposure to arsenic; using metrics such as the hazard quotient (HQ), hazard index (HI) and chronic hazard index (CHI) in arsenic-exposed communities (Kamunda et al. 2016; Ngole-Jeme and Fantke 2017; Genthe et al. 2018). However, these studies did not involve the collection of biological and environmental samples to measure arsenic exposure and identify possible pathways of this exposure.

Therefore, there is a paucity of research in South Africa pertaining to human exposure to arsenic in communities that are potentially exposed to elevated concentrations of arsenic present in environmental media. One of the few such studies related to arsenic and human health found that arsenic concentrations in maternal blood of coastal residents at delivery were correlated with self-reported type of water source for drinking water (piped or borehole) (Röllin et al. 2017). Our study, one of the first in the country, aimed to establish if arsenic-exposed communities faced higher risks of adverse health effects due to elevated blood arsenic concentrations compared to people in non-exposed villages. Other considerations included whether the possible sources of this arsenic was from water and/or soil exposure by measuring arsenic concentrations in both. We investigated long-term exposure of residents living in areas known to have high environmental arsenic concentrations in Giyani, Limpopo province. A two-pronged approach was applied by (i) analysing the blood arsenic concentrations of individuals living in exposed and non-exposed areas; and (ii) evaluating arsenic concentrations in drinking water and soil samples from participants’ dwellings and gardens respectively, to identify potential sources of arsenic exposure.

## Methods

### Study area

**Fig. 1:**
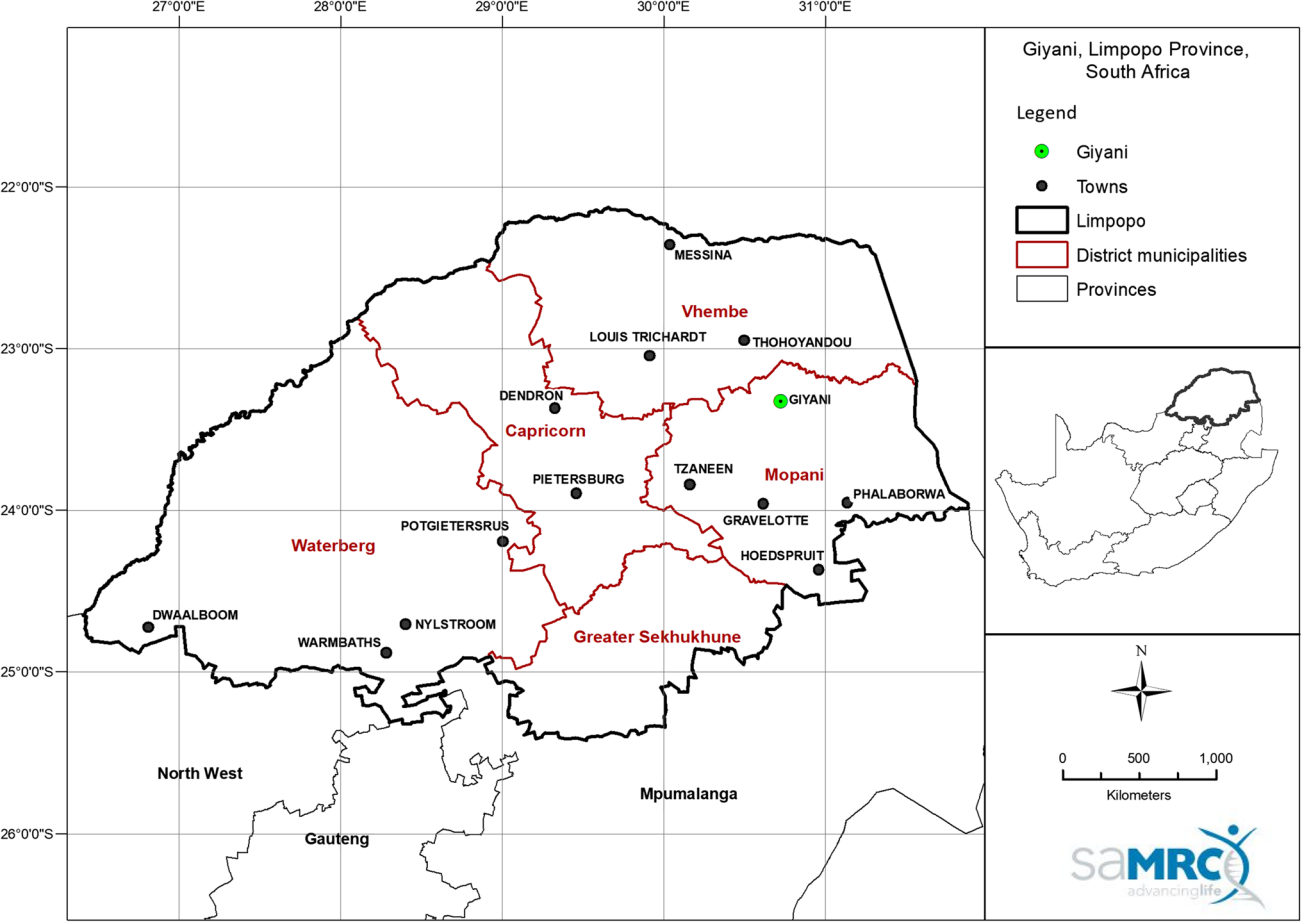
Map showing the location of Giyani in Limpopo province, South Africa

Greater Giyani is one of five local municipalities in the Mopani District Municipality in Limpopo province (Fig. 1). It is largely rural with an estimated total population of 244 217 consisting of approximately 91 villages and some semi-urban settlements (Giyani 2017). Poverty is rife in the area, with 42% of households reporting that they had no income or lived on ZAR800 per month, which is equivalent to ~ USD 51 (Giyani 2017). The study area was selected due to existing evidence of potentially high levels of arsenic in water and soil (Munyangane et al. 2017; Kapwata et al. 2020; Mudzielwana et al. 2020).

### Study design and participant recruitment

A multi-disciplinary, cross-sectional study design with exposed and control sites was used to select study participants. This epidemiological design has been used in several arsenic exposure studies (National Research Council 1999; Roh et al. 2017). The two exposed villages were selected based on findings from previous studies which measured elevated soil and water concentrations of arsenic exceeding local and international guidelines (Munyangane et al. 2017; Kapwata et al. 2020; Mudzielwana et al. 2020). The exposed villages, Maswanganyi village and Muyexe village, were categorised as ‘high’ and ‘medium/low’ exposure sites, respectively. Tomu village, which was selected as a control site, had similar demographic characteristics to the exposed villages, but studies have not found elevated concentrations of arsenic in the soil and water environments there. Moreover, there was no evidence of activities or industry to indicate potential sources of arsenic.

Based on a sample size calculation, a minimum sample size of 124 participants, of which 62 were exposed and 62 were unexposed, was required to detect a difference of 20% or greater, assuming that the prevalence of abnormal arsenic concentrations in blood in unexposed individuals is 10%. Participants recruited for the study were adults between 18 and 65 years of age and had lived in the area for more than 10 years. This age range was selected as an inclusion criterion to provide information about the susceptibility to arsenic across different age groups and to reduce loss to follow-up (Chen et al. 2011; Zhang 2016). Households and eligible participants from each home were randomly selected. The purpose of our study was verbally explained to participants prior to obtaining informed signed consent. Questionnaires were administered at the participant’s residence, and blood samples were collected from consenting participants. Water and soil from their water sources and residential garden sites were also collected.

### Questionnaire administration

Fieldworkers administered an electronic version of a structured questionnaire, which was developed using Redcap software (Harris et al. 2009). It is a secure web application used to conduct questionnaire surveys and to manage databases. The questionnaire requested information on socio-demographic information (i.e., age, level of education, employment status, employment history, sources of income and monthly household income), household characteristics (i.e., type and condition of dwelling, number of people living in the home, and fuel sources for cooking and heating), food sources (i.e., whether participants ate homegrown vegetables) and water sources (i.e., piped, borehole and river), current and past medical conditions (including chronic conditions) and behavioural practices (i.e., alcohol and tobacco use) that could be potential risk factors for arsenic exposure.

### Blood samples

Blood serves as a biomarker of recent/short-term arsenic exposure (Arcega-Cabrera et al. 2018). However, with chronic and continuing exposure, steady-state concentrations are achieved thus blood has the potential to serve as a biomarker of past exposure (Arcega-Cabrera et al. 2018). Nurses registered and accredited with the Health Professions Council of South Africa and the South African Nursing Council collected venous, whole blood samples from study participants in 6 mL trace metal-free tubes. Samples were stored in portable coolers and delivered to the laboratory, daily.

For the measurement of arsenic in blood, samples were diluted 25-fold with a diluent (Ammonia 2.5 mL; Butanol 6 mL, 0.1% Triton-X 50 µL and Ethylenediaminetetraacetic acid (EDTA) 50 µg in 500 mL deionised water). The following internal standards were also added to the diluent: Indium (25 µL), Germanium (25 µL), Scandium (25 µL), Rhodium (250 µL) and Iridium (250 µL). The instrument used for the analyses, namely an Agilent Inductively Coupled Plasma Mass Spectrometry (ICP-MS) 7 900, was calibrated with calibration standards prepared in the diluent using a multi-element custom standard (i.e., SPECTRASCAN – SS 028 226). The specifications of the instrument include a standard low-flow, Peltier-cooled sample introduction system; an ultra-high matrix introduction (UHMI) that significantly increases matrix tolerance and improves plasma robustness; a plasma and shield torch system (STS) which ensures high sensitivity and effective interference removal in helium mode; a fast, frequency-matching plasma radio frequency generator; off-axis ion lens; hyperbolic quadrupole; an orthogonal detector system (ODS) and an efficient vacuum system The concentrations of the standards for arsenic ranged from 0.1 to 50 µg/L, whilst the Limit of Detection (LOD) for arsenic in blood is < 0.2 µg/L. There are no published guidelines for the permissible levels of arsenic in blood, however, clinical and epidemiological studies recommend a threshold of 1 µg/L (Kumar et al. 2020; NRC 1999; ATSDR 2002).

### Water samples

The analytical technique used here has been used in previous studies to measure quantities of trace elements, including arsenic, in food and beverages consumed by both adults and children (Saghafi et al. 2021; Kiani et al. 2022; Karami et al. 2021).

Drinking water samples were collected from the main source used by the household, i.e., borehole, outdoor communal standpipe, indoor tap or outdoor storage tank. Samples were stored and transported at room temperature and analysed for arsenic concentration at an accredited laboratory. According to the United States Environmental Protection Agency (USEPA) guide for collecting and testing drinking water, samples do not need to be acid preserved if they are received by the laboratory within 14 days of sampling (USEPA 2016). Therefore, collected samples were delivered to the laboratory within the recommended times. An Inductively Coupled Plasma Mass Spectrometry Agilent 7 700 instrument was used for the analysis of arsenic concentration in each of the collected water samples. The specifications of the instrument include a low-flow Peltier cooled sample introduction system; electronic gas control to deliver precise control of all plasma and cell gases; a patented high matrix introduction (HMI) kit that virtually eliminates matrix suppression; a fast, frequency-matching plasma radio frequency generator; off-axis ion lens and efficient vacuum system (Agilent Technologies 2010). As part of quality control procedures, the methods used by the external laboratory, included the use of standard reference materials (SRMs) and quality control samples of known values. In addition, recoveries for the analysis were 94% for arsenic at 10 µg/L. The calibration range used was 0.01–500 µg/L and R^2^ value of the calibration was ≥ 0.995. The LOD and limit of quantification (LOQ) were 0.030 and 0.01 µg/L, respectively. Results were considered in the context of the WHO drinking water quality guideline—for arsenic in drinking water of 10 µg/L.

### Soil samples

Soil samples were collected from the within the top 10 cm of exposed soil, in accordance with US EPA operating procedures (USEPA 2014). They were transported to the laboratory at room temperature then ground by hand and oven-dried at 40 °C over a 48-h period. The soil was sieved to retain particles less than 2 mm for further analysis. The concentrations of arsenic were measured using a portable Niton XL2 XRF fluorescence (XRF) spectrometer (Niton XRF 2015). The LOD was 5 mg/kg for arsenic. Results were considered in relation to the Canadian Soil Quality Guidelines for the Protection of Environmental and Human Health of 20 mg/kg (CEPA 2007).

### Statistical analysis

Descriptive analyses of arsenic concentrations in blood, soil and water samples from the participants were performed separately for each village. These included the high-exposure village (Maswanganyi); the medium-/low-exposure village (Muyexe); as well as the control village (Tomu). The Shapiro–Wilk test was used to assess normality of arsenic concentrations. The Spearman’s rank correlation was used to assess the relationship between arsenic concentrations in blood, water and soil results in paired samples (i.e., blood, soil and water results were merged for each individual). The Wilcoxon-rank sum test was used to test for statistically significant differences between the three villages for the blood, water and soil arsenic levels.

We commenced with univariate quantile regression analysis, assessing village (as a proxy for exposure level) as a predictor of blood arsenic levels. Multivariate quantile regression was then used to evaluate the associations between blood arsenic levels and the village category (high-exposure, medium-/low-exposure and the control), whilst adjusting for potential confounders and effect modifiers including gender, house type, employment status, tobacco use, consumption of homegrown vegetables and the use of piped or borehole water for consumption and domestic uses. A variable (i.e., potential confounder) was included in the adjusted analysis if it was significantly related to either arsenic concentration, or exposed/unexposed village; or if its inclusion changed the model coefficients for exposed/unexposed village by more than 10%. All analyses were performed using Stata 15 (StataCorp 2017), and *p*-values less than 0.05 were considered statistically significant.

## Results

### Descriptive statistics

The characteristics of study participants are presented in Table 1. These characteristics are also possible determinants of behaviours and exposures that affect arsenic concentration in blood (Rahbar et al. 2022; Mathee et al. 2022). Sixty-six participants were enrolled into the study, 43 from the two arsenic exposed villages known to have high- to medium- and low-environmental arsenic levels and 23 were in the unexposed control group living in a village known to not have high levels of arsenic. More than three-quarters of participants used borehole water for domestic purposes (86%) and to irrigate vegetable gardens for homegrown food (75%) (Table 1). Subsistence farming was a common practice, with 90% of participants reporting that they consumed vegetables grown in their own gardens. Almost half of the participants were unemployed (44%), and this could have contributed to households practicing subsistence farming and growing their own food to survive.

**Table 1:** Characteristics of study participants (*N* = 65)

Variable	Category	Frequency	
		*n*	%
Age (years)	18–34	19	30
	35–59	31	49
	> 60	13	21
Gender	Male	18	28
	Female	46	72
Type of house	Formal	60	95
	Informal/traditional	3	5
Number of people in home	< 5	21	32
	5–10	35	53
	> 10	10	15
Employment status	Employed	26	50
	Unemployed	23	44
	Retired	3	6
What is the current source of drinking water for your home*	Piped water	23	35
	Borehole	57	86
	River	1	2
Do you eat vegetables grown in home soil	Yes	60	91
	No	6	9
Where do you get the water for watering your garden*	Piped water (inside dwelling and communal standpipe)	20	31
	Borehole	50	76
	River	0	0
Do you currently eat soil	Yes	3	5
	No	62	95
Do you currently use any tobacco products	Yes	3	5
	No	63	95
Have you ever been diagnosed by a doctor with the following conditions*			
Hypertension	Yes	11	17
	No	55	83
Diabetes	Yes	4	6
	No	62	94
High cholesterol	Yes	-	-
	No	66	100
Heart attack or stroke	Yes	1	2
	No	65	98
Pneumonia	Yes	1	2
	No	65	98
Bronchitis	Yes	-	-
	No	66	100
Asthma	Yes	-	-
	No	66	100
Cancer	Yes	-	-
	No	66	100
Skin lesions (i.e., cysts, bumps, lumps, rash)/skin irritation	Yes	-	-
	No	66	100
Covid-19	Yes	-	-
	No	66	100

*Participants could select more than one option

### Arsenic concentrations in water, soil and blood

**Fig. 2:**
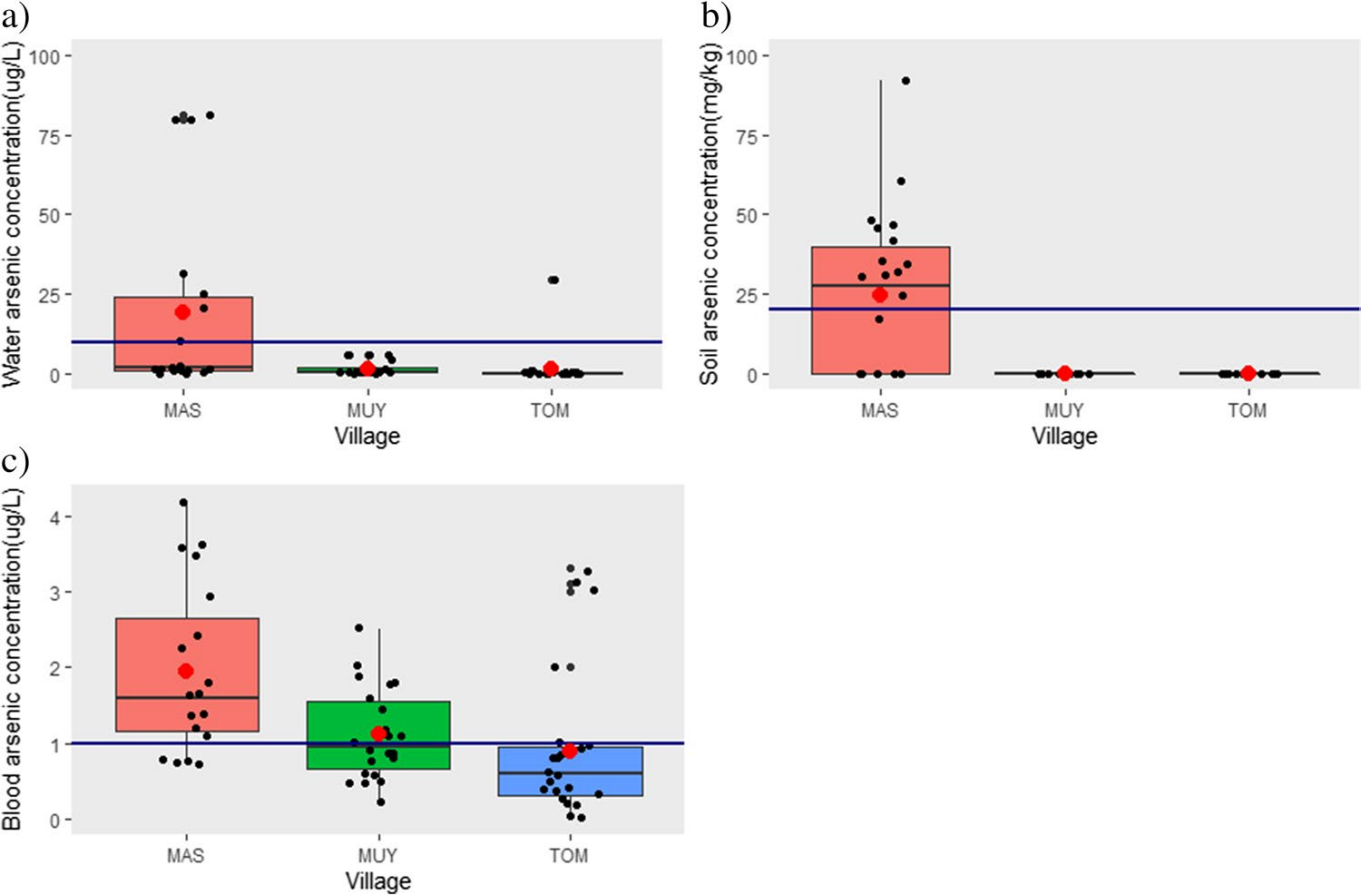
Arsenic concentrations in participants’ main source of **a** water, **b** soil from participants’ gardens and **c** participants’ blood samples in high exposure village—Maswanganyi (MAS), medium-/low-exposure village—Muyexe (MUY), and control village—Tomu (TOM)

Figure 2 presents the means, medians and the inter quartile range of arsenic concentrations for the three villages. There was a statistically significant difference in the distribution of arsenic in water samples amongst the three villages (*p*-value = 0.0002). with significant differences observed for all pairwise comparisons. Median arsenic concentrations are reported because the means were affected by high outliers that were verified with the laboratory as true values.

Elevated arsenic concentrations in water were observed in the high-exposure village of Maswanganyi village (median = 1.75 µg/L; range = 0.02 to 81.30 µg/L), followed by Muyexe, the medium-/low-exposure village (median = 0.45 µg/L; range = 0.100 to 6.00 µg/L) and Tomu, the control village (median = 0.15 µg/L; range =  < LOD to 29.30 µg/L). Thirty-nine percent of drinking water samples from the high-exposure village, had arsenic concentrations that were above the WHO drinking water quality guideline value for arsenic in drinking water (WHO 2017) (see supplementary Table S1).

There was a statistically significant difference in the distribution of arsenic concentrations in soil, amongst the villages (*p*-value = 0.0003). The median and range of arsenic concentrations in soil were 23.91 mg/kg and < LOD to 92.10 mg/kg, respectively. More than half (55%) of the soil samples collected from the high-exposure village, exceeded the recommended value of 20 mg/kg from the Canadian Soil Quality Guidelines for the Protection of Environmental and Human Health (CEPA 2007). Arsenic concentrations in all soil samples collected from participants’ gardens in the medium-/low-exposure and control villages were below the LOD.

There was also a statistically significant difference in the distribution of arsenic in blood amongst participants living in the villages (*p*-value = 0.0005). In the high-exposure village, arsenic concentrations amongst participants ranged from 0.7 to 4.2 µg/L with a median of 1.6 µg/L; in the medium-/low-exposure village, concentrations ranged from < LOD to 2.5 µg/L with a median of 0.90 µg/L whilst in the control village, arsenic levels ranged from < LOD to 3.3 µg/L. Almost 80% of blood samples collected from participants in the high-exposure village had arsenic concentrations above the recommended threshold of 1 µg/L (ATSDR 2002) whilst 45% of blood samples from the medium-/low-exposure group exceeded this threshold (Table S1). Only 19% of blood arsenic concentrations from participants in the control village were above the level of concern.

### Relations between environmental variables and blood arsenic concentrations

There were significant positive correlations between arsenic in blood and (Spearman’s rho = 0.391; *p*-value = 0.0015), specifically (Spearman’s rho = 0.292; *p*-value = 0.031). There was also a statistically significant positive association between arsenic concentrations in participants’ blood and soil collected from their gardens (Spearman’s rho = 0.290; *p*-value = 0.051).

The key exposure variable in our study was ‘village’ and it had three levels—high-exposure (Maswanganyi), medium-/low-exposure (Muyexe) and the control site (Tomu)—which was also the reference category in the regression. Univariate quantile regression analysis found that participant blood arsenic concentrations increased by 0.034 µg/L (95% CI = 0.02–0.05), for each one unit increase in matching water arsenic concentrations (*p*-value < 0.001) (Table S2a). However, participant blood and soil concentrations were not significantly associated (Table S2b). We also observed that individuals from the high-exposure village were associated with significantly higher blood arsenic levels, than individuals in the control site (coefficient = 1; 95% CI = 0.36–2.76; *p*-value = 0.007). However, individuals in the medium-/low-exposure site did not have significantly different blood arsenic concentrations, compared to individuals from the control village (coefficient = 0.3; 95% CI =  − 0.39 to 0.99; *p*-value = 0.387).

For the multivariate regression analysis, we compiled a list of potential confounders for the relationship between exposure (i.e., ‘village’) and arsenic concentration in blood using existing literature for effects of borehole water, homegrown vegetables and age on arsenic in blood (Hall et al. 2006; Iyer et al. 2016; Rahbar et al. 2012; Bibi et al. 2015; Sekhar et al. 2003). Therefore, the variables included in the regression model were exposed/unexposed village, age, borehole water as main source of water, and consumption of homegrown vegetables (Table 2). After adjusting for age, water source and homegrown vegetable consumption, participants from the high-exposure village had significantly higher blood concentrations than those from the control village (Coefficient 1.00; 95% CI = 0.25–1.74; *p*-value = 0.009). These results concur with our initial hypothesis that the community of Maswanganyi likely experience higher arsenic exposure compared to residents of Tomu village which was the unexposed site.

**Table 2:** Results of multivariate quantile regression model assessing the association between study participant characteristics and blood arsenic concentrations. The unexposed village (control) was Tomu

	Coefficient	*p*-value	95% CI*
Village name (exposure category)			
Muyexe (medium-/low-exposure)	0.201	0.566	− 0.497 to -0.9
Maswanganyi (high-exposure)	1.000	0.009	0.254–1.746
Age	0.000	0.386	0.000–0.000
Drinking borehole water	− 0.202	0.639	− 1.058 to 0.655
Consuming homegrown vegetables	− 0.498	0.362	− 1.584 to 0.588

*CI = 95% simultaneous confidence band for the quantile coefficient estimation

## Discussion

Arsenic contamination of water and soil has been investigated globally, given that arsenic is a known human carcinogen and has numerous adverse health effects. A limited amount of research has been undertaken in African countries, including South Africa, to assess arsenic exposure to contaminated water and/or soil, most of which have been desk-top human health risk assessment studies (Rosas-Castor et al. 2014; Affum et al. 2015; Wang et al. 2019; Bortey-Sam et al. 2015). Our study is considered the first amongst (South) African studies that analysed arsenic concentrations in water and soil, and took blood samples from individuals living in arsenic-exposed and non-exposed areas to quantitatively consider individual arsenic exposure.

Arsenic concentrations in water from our hypothesised high arsenic exposure site were above the WHO guideline value for arsenic in drinking water of 10 µg/L (WHO 2017). The WHO implemented this provisional guideline value taking into consideration the Joint Food and Agriculture Organization of the United Nations (FAO)/WHO Expert Committee on Food Additives (JECFA) evaluations of literature. They concluded that for certain regions of the world with concentrations of arsenic in drinking water that ranged between 50 to100 µg/L, evidence of adverse health effects existed. In other regions, where arsenic concentrations were between 10 and 50 µg/L, the possibility of adverse effects persisted, but would likely be at a low incidence (WHO 2018). Our study provided evidence of elevated arsenic levels in drinking water in the highly exposed village, with 39% of water samples exceeding the WHO recommendation and placing residents at risk of adverse health outcomes. For example, a large survey of Wisconsin residents who consumed groundwater with arsenic concentrations of 10 µg/L for 20 or more years had increased prevalence of chronic cardiac diseases and a high incidence of cardiac bypass surgery and depression (Zierold et al. 2004). Furthermore, a study using data from one of the largest populations at risk of arsenic exposure due to contaminated groundwater showed more than 24 000 adult deaths annually could be attributed to arsenic exposures of 10–50 µg/L (Flanagan et al. 2012).

The analysis of soil samples showed that arsenic concentrations in the control and medium–low exposure sites were below the LOD. However, high concentrations were found in soil samples from the high-exposure village. We also found a significant correlation between participant’s blood arsenic concentrations and matching soil arsenic concentrations; a relationship which is supported by other studies (Madrid et al. 2022; Li et al. 2021). Almost 90% of study participants reported that they consumed vegetables grown in the gardens from which soil samples were collected. Therefore, in addition to drinking water, the transfer of arsenic from soils to the edible parts of plants is another possible route of arsenic entry into the human body (Gebeyehu and Bayissa 2020).

As anticipated, the trend of blood arsenic concentrations showed that the highest levels were observed in the high-exposure site where almost 80% of blood samples exceeded the recommended threshold of 1 µg/L (ATSDR 2002). Similarly, 45% of samples from the medium-/low-exposure site exceeded the recommended threshold of 1 µg/L, whilst only 19% of samples from the control were above the threshold. Arsenic. Skin lesions were reported in about 23% of respondents with an arsenic blood range of 1.6–5.4 µg/L from a cohort study in Bangladesh exposed to high levels of arsenic from groundwater (Hall et al. 2006). The detection of arsenic in blood samples from non-exposed residents could have been caused by participants smoking tobacco products, arsenic is one of the chemical compounds found in cigarettes (WHO 2009). Although only a small percentage of respondents reported using tobacco products, the true prevalence of smoking could be higher as smoking status is often underreported due to stigma (Singh et al. 2022; Liber and Warner 2018).

Our univariate regression results reinforced the finding that the study participants from the high-exposure village had significantly higher blood arsenic concentrations than those participants from the control site. This was still true after adjusting for potential confounders such as age, water source and homegrown vegetable consumption in the multivariate regression. Therefore, our findings show that blood may be a useful biomarker for exposure to arsenic-contaminated water and soil. Previous studies also suggest blood arsenic concentrations are a reliable indicator of chronic, continuing exposure (Hall et al. 2006; McClintock et al. 2012).

A significant proportion of participants (86%) used borehole water. We found a significant correlation between participants’ blood arsenic concentrations and matching borehole water arsenic concentrations similar to other studies around the world (Katiyar and Singh 2014; Kumar et al. 2020; Rodrigues et al. 2016; Arikan et al. 2015; Arcega-Cabrera et al. 2017). Groundwater extracted via borehole or hand-dug wells may be contaminated with arsenic due to the hydrogeochemistry of the environment, for example arsenic present in coal in India (Patel et al. 2012), or from an anthropogenic source (Mudzielwana et al. 2020). Previous studies have found that arsenic contamination of drinking water sources can occur because of proximity to naturally-occurring arsenic found in certain types of bedrock and sediments. Whilst we did not ask participants their reasons for relying on borehole water as their main water source, which was identified as a shortcoming of our research, several reasons may be surmised. Water supply as a basic service is oftentimes unreliable in rural areas in Limpopo with frequent disruptions in supply (Nguyen et al. 2021; Luvhimbi et al. 2022; Majuru et al. 2012). Another consideration may be that surface water from rivers or dams may be deemed less suitable for human consumption; in terms of quality compared to groundwater (Madilonga et al. 2021). Using groundwater when it is more readily available than other sources, such as piped water supply, is thus a necessity but also introduces an issue of environmental injustice. There is no easy, low-cost solution at household or community level to remove arsenic from water. Therefore, it is imperative that appropriate policy implementation actions are taken to ensure safe drinking water is made available to all individuals and villages at risk of arsenic exposure via water, in particular from groundwater sources.

### Study limitations

Several study limitations were considered. The timing of water sampling for the study was likely affected by season, since concentrations of chemicals in water may be diluted during the rainy season compared to the dry season, and additional samples, taken during different seasons may be required to supplement these findings. The minimum sample size of individuals required for the study was not met due to reluctance from potential participants to provide a blood sample due to traditional and cultural beliefs. There was also community unrest during the time of fieldwork caused by leadership contestations, and therefore, it was unsafe to proceed with data collection in some areas. Whilst participants were asked what their main water source was, multiple water sources could have been used in addition to their self-reported main source. Future studies should consider asking for the main water sources as well as secondary water sources.

## Conclusions

Arsenic is naturally and anthropogenically present at high levels in groundwater in several countries around the world. Contaminated borehole water used for drinking and irrigation of food crops poses a public health threat. We assessed arsenic concentrations in study participants’ main water source, soil from their garden and participants’ blood samples. To the best of our knowledge, this is one of the first studies to analyse arsenic in blood at the individual level in Limpopo province, South Africa. We found significant associations between arsenic concentrations in borehole water and blood arsenic concentrations. This was particularly concerning because most of our study participants relied on borehole water as their main source of drinking water. In rural settings, such as the villages in our study, lack of access to piped water leads to a reliance on borehole water. Service provision of potable water for consumption, food preparation and irrigation of food crops in areas with arsenic-contaminated water needs to be improved to protect public health. Soil arsenic concentrations was correlated with blood arsenic concentrations, indicating possible exposure to arsenic through ingestion of homegrown produce. Further work is required to investigate arsenic concentrations in these plants and vegetables. We also found that study participants from the high-exposure village had significantly higher blood arsenic concentrations than those from the control site, thus demonstrating that blood arsenic is potentially a good biomarker of human arsenic exposure.

### Supplementary Information

Below is the link to the electronic supplementary material.

Supplementary file1 (DOCX 18 KB)

## Data Availability

The datasets generated during this study are available from the authors upon request.
