# Correction to: Relations between personal exposure to elevated concentrations of arsenic in water and soil and blood arsenic levels amongst people living in rural areas in Limpopo, South Africa

**DOI:** 10.1007/s11356-024-32103-9

**Published:** 2024-01-30

**Authors:** Thandi Kapwata, Caradee Y. Wright, Tarylee Reddy, Renee Street, Zamantimande Kunene, Angela Mathee

**Affiliations:** 1 Environment and Health Research Unit, South African Medical Research Council, Johannesburg, 2028 South Africa; 2 Environmental Health Department, Faculty of Health Sciences, University of Johannesburg, Johannesburg, 2028 South Africa; 3 Environment and Health Research Unit, South African Medical Research Council, Pretoria, 0084 South Africa; 4 Department of Geography, Geoinformatics and Meteorology, University of Pretoria, Pretoria, 0001 South Africa; 5 Biostatistics Research Unit, South African Medical Research Council, Durban, 4001 South Africa; 6 School of Mathematics, Statistics and Computer Science, University of KwaZulu Natal, Pietermaritzburg, 3201 South Africa; 7 Environment and Health Research Unit, South African Medical Research Council, Durban, 4001 South Africa; 8 School of Public Health, University of the Witwatersrand, Johannesburg, 2028 South Africa

**Correction to: Environmental Science and Pollution Research (2023) 30:65204–65216**

10.1007/s11356-023-26813-9

The correct presentation of affiliation 4 is modified in the original published proof.

The Original article has been corrected.

