# Supplementary material for: Relations between personal exposure to elevated concentrations of arsenic in water and soil and blood arsenic levels amongst people living in rural areas in Limpopo, South Africa
Source: Environ Sci Pollut Res Int. 2023 Apr 20;30(24):65204–16. doi: 10.1007/s11356-023-26813-9 (PMC10116462; doi:10.1007/s11356-023-26813-9)
Supplement: Supplementary file 1 — Supplementary file1 (DOCX 18 KB) [file 11356_2023_26813_MOESM1_ESM.docx]

Table S1 (a) and (b): Water (a), soil (b) and blood (c) samples divided into categories of arsenic concentrations

(a)

|  |  | Water concentration (ug/L) | | |
| --- | --- | --- | --- | --- |
|  |  | <5 | 5 – 10 | >10 |
| Village | Mas (n=18) | 11 (61.11%) | – | 7 (38.89%) |
|  | Muy (n=22) | 18 (81.82%) | 4 (18.18%) |  |
|  | Tom (n=22) | 21 (95.45%) | – | 1 (4.55%) |
| (b) |  |  |  |  |
|  |  | Soil concentration (mg/kg) |  |  |
|  |  | <10 | 10 – 20 | >20 |
| Village | Mas (n=22) | 9 (40.91%) | 1 (4.55%) | 12 (54.55%) |
|  | Muy (n=27) | 27 (100%) | – | – |
|  | Tom (n=25) | 25 (100%) | – | – |

(c)

| Blood concentration (ug/L) | | | |
| --- | --- | --- | --- |
|  |  | <1 | >1 |
| Village | Mas (n=19) | 4 (21.05%) | 15 (78.95 %) |
|  | Muy (n=22) | 12 (54.55%) | 10 (45.45%) |
|  | Tom (n=21) | 17 (80.95%) | 4 (19.05%) |

Table S2 (a) and (b): Results of quantile regression models evaluating associations between blood and (a) water and (b) soil arsenic concentrations

(a)

| blood | Coefficient | p-value | 95% CI* |
| --- | --- | --- | --- |
| water | 0.03 | 0.00 | 0.02 – 0.05 |
| _cons | 0.80 | 0.00 | 0.55 – 1.04 |

*CI = Confidence interval

(b)

| blood | Coefficient | p-value | 95% CI |
| --- | --- | --- | --- |
| soil | 0.02 | 0.14 | -0.0054 – 0.04 |
| _cons | 0.80 | 0.001 | 0.34 – 1.26 |
